# Maternal mental health and children’s development: a bi-directional relationship?

**DOI:** 10.23889/ijpds.v7i3.1937

**Published:** 2022-08-25

**Authors:** Natalie Johnston, Elizabeth Ford, Richard Tyler, Vicki Spencer-Hughes, Anotida Madzvamuse, Graham Evans, Kate Gilchrist, Jacqueline Clay

**Affiliations:** 1 Brighton & Hove City Council; 2 Brighton and Sussex Medical School; 3 West Sussex County Council; 4 East Sussex County Council; 5 University of Sussex

## Objectives

Public health intelligence teams in Sussex wanted to use newly linked health and social care data, to gain insights into local patterns of multi-morbidity, service use, service provision and socio-demographic data. In this study we report initial exploration of this new linked dataset, in a partnership between university and local authority analysts.

## Approach

The Sussex Integrated Dataset (SID) comprises person-level health and social care data on residents and services users across Sussex. During a 6-month secondment, two analysts evaluated the number of data sources available for each individual, evaluated data quality for identifying long-term conditions, developed presentation methods to compare SID outputs on demographics and condition prevalence with open source or expected distributions, and identified the skills-mix and infrastructure required in local authorities for future work. They worked alongside the SID data processing team to inform and improve data quality; and with university data-scientists to learn prediction modelling.

## Results

Analysts established an efficient querying system to investigate the breadth of data available, more thoroughly focusing on encounters and demographic data in all sources. Long-term conditions were identified through code lists in a range of NHS data sources, to enable consideration of multi-morbidity by demographic. A range of quality issues were identified, such as non-current patients being uploaded into the SID, distorting prevalence estimates, and GP practice populations that did not match expected figures published by NHS digital. Results were represented in multi-morbidity plots, maps, and theographs. Through this data exploration, we have been able to identify the skills-mix needed for local Public Health Intelligence teams to maximise the use of linked data to achieve Public Health objectives.

## Conclusion

We have made many conceptual breakthroughs, particularly in understanding data quality, however still need a more complete understanding of quality issues in SID for public health outputs to have meaningful use. Further investigation into the patterns of service use, as well as modelling of multi-morbidity to make predictions and target interventions, will be key next steps.

